# 

**Published:** 1893-11-04

**Authors:** 


### CONTENTS.

**Consultants and General Practitioners** - - - - - 65
**Hospitals in India.**—I. The Bengal Presidency - - - - 66
**Injection of Hydrogen Peroxide into the Peritoneal Cavity.**—By Sir B. W. Richardson, M.D., F.R.S. - - - 67
**The Medical Work of the Local Government Board.**—The Incidence of Disease and Sanitation - - - - - - - 68
**On the Cellular Structure of Living Organisms.**—II. - 69
**The Field of Science** - - - - - - - - - - 70
**Annotations.**—Indian Oculists, British Jurors, and the Medical Council—Evening Out-Patient Departments—Is Alcohol a Food? 71
**Notes and News** - - - - - - - - - - 72
**Editor's Letter-Box.**—The General Infirmary, Leeds—The General Hospital, Birmingham—West Ham Hospital, Stratford, E.—Cruelty in Irish Parents - - - - - - - - 79
**The Hospital Clinic**—
Royal Infirmary Edinburgh.—The Treatment of Hare Lip - 73
Some Dangers Attending the Use of the Rectangular Position in Injuries of the Elbow-joint. By A. Hanbury Frere, M.B. - - - - - - - - - - 74
New Drugs and Preparations —Borax and Bromides in Epilepsy 76
**The Institutional Workshop**—
Hospital Administration. Some Metropolitan, Provincial, Scotch, and Irish Special Hospitals Compared - - - - 77
Construction Notes - - - - - - - - - 78
**Practical Departments.**—Washstands - - - - - - 80
**Scraps and Gleanings** - - - - - - - - - 80
**The Book World of Medicine and Science** - - - - vii
**Medical Vacancies and Appointments** - - - - - ix

### EXTRA SUPPLEMENT.—THE NURSING MIRROR.

**News from the Nursing World.**—"Wait for an Answer"—Ask the Doctor—Not All by Rule—Is there any Charge?—Women Workers—Nurses' Home and Club—At Last—A Sad Tragedy—Indian Hospitals—A Nurse's Birthday—Short Items xli
**On the Nursing of Diseases of the Nervous System.**—V. Causes of Paraplegia - - - - - - - - xliii
**Council Meeting of the R.B.N.A.** - - - - - - xliii
**Nursing in the United States.**—Proper Organisation of Training Schools. By Miss Darche - - - - - - xliv
**Nursing in Newfoundland.**—Mission to Deep-Sea Fishermen—Labrador - - - - - - - - - xlv
**Nursing in Japan** - - - - - - - - - xlv
**A Bird Show** - - - - - - - - - - xlvi
**Women's Work.** I.—The Choice of a Career - - - - xlvi
**Where to Go** - - - - - - - - - - xlvii
**Notes and Queries—Appointments** - - - - - xlviii
**For Reading to the Sick.**—Misunderstanding - - - xlvii
**Everybody's Opinion.**—Nursing at Johannesburg - - xlvii
**Wants and Workers** - - - - - - - - - xlvii
**A Groat's Worth of Wit.**—On Short Stories - - - - xlviii
**Novelties for Nurses** - - - - - - - - xlviii
**The Book World for Women and Nurses** - - - - xlix

